# Reduced dynamical variability in depression: evidence from EEG and QIF-E network simulation

**DOI:** 10.3389/fncom.2026.1899952

**Published:** 2026-08-27

**Authors:** Gabriel Moreno Cunha, Gisele Santana, Jose Garcia Vivas Miranda, Gilberto Corso, Thaise Graziele L. de O. Toutain, Marcelo M. S. Lima, Matheus Phellipe Brasil de Sousa, Gustavo Zampier dos Santos Lima

**Affiliations:** 1 Modelling and Neurodynamics Simulation Laboratory, Universidade Federal do Rio Grande do Norte, Natal, Brazil; 2 Theoretical and Experimental Physics Department, Universidade Federal do Rio Grande do Norte, Natal, Brazil; 3 Institute for Neural Information Processing, University Medical Center Hamburg-Eppendorf (UKE), Hamburg, Germany; 4 Physics Institute, Universidade Federal da Bahia, Salvador, Brazil; 5 Biophysics Department, Universidade Federal do Rio Grande do Norte, Natal, Brazil; 6 Institute of Health Sciences, Universidade Federal da Bahia, Salvador, Brazil; 7 Laboratory of Neurophysiology, Department of Physiology, Universidade Federal do Paraná, Curitiba, Brazil; 8 Science and Technology School, Universidade Federal do Rio Grande do Norte, Natal, Brazil

**Keywords:** complexity, electric communication, electroencephalography, Major Depressive Disorder, modeling, multiscale entropy, small-world network, variability

## Abstract

Alterations in neural signal complexity have been consistently reported in Major Depressive Disorder (MDD), suggesting changes in the underlying dynamics of brain activity. In this study, we investigate whether changes in neural signal complexity observed in MDD can be characterized using multiscale entropy (MSE) analysis, a method that quantifies temporal complexity across multiple scales. We analyzed electroencephalographic (EEG) recordings from individuals diagnosed with MDD and healthy controls, and compared these results with simulations of neural network models incorporating different forms of local electrical coupling. The EEG analysis revealed significant alterations in signal complexity in MDD, characterized by higher mean entropy at broader time scales and reduced inter-individual variability when compared to healthy controls. To explore potential dynamical mechanisms underlying these observations, we employed a Quadratic Integrate-and-Fire (QIF) neuronal network model with tunable local electrical coupling and small-world synaptic topology. Network topology and coupling parameters were systematically varied to assess their impact on signal complexity, without assuming a direct physiological correspondence between model components and specific biological mechanisms. MSE was used to quantify the irregularity and predictability of both empirical EEG signals and simulated network activity across multiple temporal scales. We found that network configurations lacking local electrical coupling reproduced key entropy features observed in the EEG signals of individuals with MDD, including increased mean entropy and reduced dispersion across realizations. In contrast, simulations with local electrical coupling exhibited lower average entropy and greater variability, resembling the entropy patterns observed in healthy control EEG data. These results suggest that differences in local coupling structure can modulate the balance between complexity and variability in network dynamics, potentially influencing the range of accessible dynamical states. Rather than establishing causality, this comparative analysis highlights how simplified models of local electrical coupling can phenomenologically account for entropy alterations observed in MDD, providing a computational framework for exploring links between network dynamics and large-scale brain signal complexity.

## 1 Introduction

Neuroscience has increasingly relied onmathematical models and simulations to elucidate the complex dynamics of the brain (Breakspear, 2017; Dayan and Abbott, 2005). By employing mathematical approaches, researchers can explore how the structural organization of neural networks influences brain function and dynamics. Neural network topology mainly describes the arrangement of synaptic connections in the brain (Park and Friston, 2013), while recent studies have suggested that non-synaptic forms of local electrical interaction may also influence neural dynamics (Anastassiou et al., 2011; Pinotsis and Miller, 2023; Cunha et al., 2022, 2023; Ruffini et al., 2020).

The relevance of neuronal network models lies in their ability to bridge the gap between microscopic synaptic mechanisms and macroscopic brain dynamics, offering insights into both normal cognitive processes and pathological states (Krejcar and Namazi, 2025; Wright and Liley, 1996). Although alterations in synaptic transmission, excitation-inhibition balance, and neuromodulatory systems have been extensively investigated in computational models of Major Depressive Disorder (MDD) (Belujon and Grace, 2017; Duman et al., 2019; Veeraiah et al., 2014), considerably less attention has been given to local electrical interactions between neurons, despite growing evidence that endogenous electric fields can influence neuronal synchronization and collective activity (Anastassiou et al., 2011; Anastassiou and Koch, 2015; Pinotsis and Miller, 2023; Cunha et al., 2022, 2023). From the perspective of dynamical systems, coupling strength is one of the fundamental control parameters governing collective network behavior and may substantially influence neural synchronization, stability, and signal complexity (Liu et al., 2022; Hormuzdi et al., 2004; Park and Friston, 2013). Therefore, we selected electrical coupling as the variable of interest because it represents a largely unexplored mechanism capable of modulating collective neural dynamics. Our objective is not to propose altered electrical coupling as the physiological cause of Major Depressive Disorder, but rather to investigate whether modulation of this single mechanism is sufficient to phenomenologically reproduce patterns of neural complexity observed in experimental electroencephalographic (EEG) recordings.

In this work, we investigate whether alterations in neural signal complexity observed in Major Depressive Disorder (MDD) can be phenomenologically reproduced by simplified neuronal network models incorporating different forms of local electrical coupling. Although we refer to this interaction as “ephaptic coupling” following the QIF-E framework, it should be interpreted here as a generalized form of local electrical interaction that could encompass various non-synaptic mechanisms, such as endogenous electric fields or other forms of field effects. In this work, we applied a qualitative comparison between the Quadratic Integrate-and-Fire model with electrical coupling (QIF-E) and physiological data under MDD to explore dynamical correspondences in neuronal communication under physiological disorders.

MDD is a prevalent and debilitating mental health condition that affects millions of individuals worldwide (Belmaker and Agam, 2008; Uhlhaas and Singer, 2012). Its relevance today is underscored by its association with reduced quality of life, increased healthcare costs, and heightened risks of comorbidities such as anxiety and cardiovascular diseases (Yorbik et al., 2004). Neuroimaging studies show that people with MDD have reduced gray matter in the right dorsolateral prefrontal cortex (DLPFC) (Zheng et al., 2021). At the biochemical level, MDD is associated with dysregulation of monoaminergic systems, particularly serotonin, dopamine, and norepinephrine (Belujon and Grace, 2017). Moreover, MDD is not only associated with deficits in monoaminergic neurotransmitters, but also with impaired glutamatergic and GABAergic signaling (Duman et al., 2019; Veeraiah et al., 2014). In addition, individuals with MDD exhibit altered prefrontal cortex function, reflected by reduced glucose metabolism and cerebral blood flow (Ernst et al., 2017; Li et al., 2017). These changes are associated with symptoms such as persistent sadness, anhedonia, and cognitive deficits (Drevets et al., 2008). Understanding large-scale neural dynamics in MDD is therefore an important step toward characterizing the disorder at the systems level.

Neural communication occurs not only through direct synaptic transmission but also via collective oscillatory dynamics and cross-frequency interactions (Voytek and Knight, 2015; Lewis et al., 2016; Bullock, 1997). Local electrical interactions mediated by the extracellular space may contribute to synchronization and modulation of neural activity, influencing collective network dynamics (Anastassiou and Koch, 2015; de Sousa et al., 2024). Such interactions have been proposed as potential modulators of the balance between variability and stability in neural systems (Pinotsis and Miller, 2023; Guo and Ong, 2024; Rabinovitch et al., 2024; Moreno Cunha et al., 2024). Brain signal complexity emerges from the interaction of multiple coupling mechanisms operating across spatial and temporal scales (Moreno Cunha et al., 2024).

The neural network dynamical theory can model how neural activity adapts to changes in network topology and coupling strength (Liu et al., 2022; Hormuzdi et al., 2004). Several studies have reported alterations in neural complexity in MDD, motivating the use of computational models to explore how changes in coupling structure may influence large-scale signal properties. Therefore, QIF-E simulations provide an alternative dynamical framework for understanding disorder-related neural dynamics, such as MDD.

The present study compares qualitatively the Multiscale Entropy (MSE) results obtained from the QIF-E Local Average Potential (LAP) and the real Electroencephalogram (EEG) data under MDD. The main results provide an interesting insight into brain disorders concerning the network dynamical balance. The present work is organized as follows: The Methods section describes the EEG dataset, the neuronal network model, and the MSE framework. The Results section presents the entropy profiles across multiple time scales for both empirical and simulated signals. Finally, the Discussion addresses the implications and limitations of the phenomenological correspondence observed between data and model.

## 2 Methods

This study combines the analysis of empirical EEG recordings with computational modeling to compare neural signal complexity across experimental and simulated data. The first component focuses on the characterization of signal complexity in EEG recordings obtained from healthy individuals (Control Group, CG) and individuals diagnosed with MDD (MDD Group, MG). The second component consists of *in silico* simulations of neuronal network activity, used as a phenomenological reference to explore potential dynamical correlates with empirical EEG results.

The model parameters and network topology were systematically varied to explore their impact on signal complexity, without assuming a direct correspondence with specific physiological mechanisms. In addition, we analyzed the role of local electrical coupling into the simulated network and compared the statistical results of MSE obtained from the simulations with real EEG data. The MSE obtained from simulated signals was compared with that derived from EEG data to assess phenomenological similarities rather than biological identity in signal complexity patterns.

### 2.1 Subjects and experimental set-up

The EEG data analyzed in this work were obtained from a previously published and publicly available dataset, comprising 30 individuals diagnosed with MDD (MDD Group, MG) and 28 healthy individuals (Control Group, CG). The MG had a mean age of 40 years (σ = ±12), while the CG group had a mean age of 38 years (σ = ±15). Participants were originally recruited at the Hospital University Sains Malaysia (HUSM), Malaysia. A senior psychiatrist from the institution confirmed the diagnosis.. Both groups were instructed to abstain from smoking and the consumption of stimulants, such as caffeinated beverages, to minimize potential confounding effects. In addition, to perform the washout procedure, individuals in the MG were required to discontinue any medication for at least 2 weeks prior to participating in the study. A formal diagnosis of major depressive disorder was used as an inclusion criterion for the MG. Exclusion criteria included the presence of psychotic symptoms, pregnancy, smoking, alcoholism, or epilepsy. Varying levels of depression severity, classified as mild, moderate, or severe, were observed among participants in the MG. The CG group was composed of individuals without any history of physical or mental disorders, verified through a screening process conducted at HUSM. Ethical approval for the study was obtained from the HUSM ethics committee, and all participants provided written informed consent. Additional details regarding recruitment, diagnosis, and ethical procedures can be found in the original publication by Mumtaz et al. (2017).

#### 2.1.1 Data collection

This study makes use of a publicly available EEG dataset, hosted on figshare under the title “*MDD Patients and Healthy Controls EEG Data (New)”* (Mumtaz et al., 2017). The dataset includes three experimental conditions for both groups: eyes closed (5 min), eyes open (5 min), and a P300 task (10 min). In this study, only the eyes-closed, 5-min recordings were analyzed, as they provide a representative resting-state condition. EEG recordings were conducted using a Brain Master Systems amplifier with a sampling rate of 256 Hz, a band-pass filter ranging from 0.5 Hz to 70 Hz, and a 50 Hz notch filter. Data were acquired using 21 electrodes (standard 10–20 system locations). Auricular electrodes were used as reference during acquisition (Mumtaz et al., 2017).

#### 2.1.2 Data processing

Data processing was performed using EEGLAB^®^ in conjunction with MATLAB^®^ (The MathWorks, Inc. Natick, Massachusetts, EUA). Initially, auricular electrodes were removed, resulting in 19 channels, which were re-referenced to the Cz electrode. Subsequently, the Cz channel was also excluded, leaving 18 channels for further analysis. The EEG signals were filtered using a band-pass filter from 0.5 Hz to 48 Hz and segmented into epochs of 1.05 s. This epoch length was chosen as a compromise between approximate signal stationarity and sufficient data length for reliable MSE estimation. Artifacts associated with ocular movements or muscle activity exceeding a threshold of ±70μV were automatically detected and removed. A subsequent visual inspection was performed to ensure data quality and remove remaining contaminated segments. After preprocessing, the number of valid epochs ranged from 168 to 286 per subject, corresponding to total analyzed durations between approximately 3 and 5 min (Toutain et al., 2023; Sallum et al., 2025).

### 2.2 Firing neuron model with local electrical coupling

The Quadratic Integrate-and-Fire Ephaptic (QIF-E) model (Cunha et al., 2022, 2023; Moreno Cunha et al., 2024) is a simplified neuron model belonging to the class of integrate-and-fire neurons, originally proposed in the literature as a phenomenological framework to investigate how local field effects, often referred to as ephaptic interactions, emerge through electrical potential differences between neighboring neurons. In this formulation, these effects are represented through a linear electrical coupling term that captures voltage-mediated interactions between neurons at the membrane level.

In contrast to physiologically detailed but computationally expensive neuronal models, the QIF-E model reproduces action-potential-like dynamics while neglecting microstructural details. Following (Moreno Cunha et al., 2024), the QIF-E model has been employed as a reduced phenomenological framework to explore how electrical interactions between neurons may influence collective dynamics, without aiming at a full biophysical description of specific mechanisms. The QIF-E model is given by


$$\begin{array}{l} \;C_{m}{\dot{V}}_{m}\left(t\right)=\underset{\text{Dynamic term}}{\underset{︸}{a.V_{m}^{2}\left(t\right)+b.V_{m}\left(t\right)}}\\ +\;\underset{\text{Synaptic Current}}{\underset{︸}{I_{0}\left(t\right)}}-\;\underset{\text{Local Electric Current}}{\underset{︸}{I_{E}\left(t\right)}}, \end{array}$$ (1)

where *C*_*m*_ is the membrane capacitance, *V*_*m*_ is the membrane potential, *a* and *b* are parameters related to the intrinsic electrical properties of the neuron membrane, and the current terms represent external and interaction-driven inputs.

In the QIF-E framework, neurons are approximated as point-like units. The electrical coupling term is derived from an equivalent circuit approximation of the membrane, following the original formulation of the model (Cunha et al., 2022, 2023; Moreno Cunha et al., 2024).

Within this phenomenological approximation, the electrical coupling term is expressed as $I_{E}^{\left(i\right)}\left(t\right)=c_{\left(j\right)}\left(V_{m}^{\left(i\right)}\left(t\right)-V_{m}^{\left(j\right)}\left(t\right)\right)$, where this term represents the transmembrane current induced in neuron (i) by the voltage difference relative to neuron (j). The coupling coefficient *c*_(*j*)_ is a phenomenological parameter that aggregates the effects of the membrane, extracellular electrical characteristics, and the distance between elements (*i*) and (*j*) into a single effective constant for each pair in the network (Holt and Koch, 1999; Logothetis et al., 2007; Anastassiou et al., 2011; Cunha et al., 2022, 2023; Moreno Cunha et al., 2024); this means that this constant is distinct for each pair of neurons.

In addition, the synaptic input is represented by the term $I_{0}^{\left(i\right)}\left(t\right)$. In this work, synaptic interactions are modeled using the CUBA (Current-Based) framework (Moreno Cunha et al., 2024; Roth and van Rossum, 2009), which assumes exponentially decaying synaptic currents: $I_{0}^{\left(i\right)}\left(t\right)=\overset{N}{\underset{k\neq i}{\sum }}\omega^{\left(k\right)}\exp\left(-\left(t-t_{0}^{\left(k\right)}\right)/\tau \right)$, where ω^(*k*)^ denotes the synaptic strength between neurons *i* and *k*, and $t_{0}^{\left(k\right)}$ corresponds to the firing time of the presynaptic neuron. The parameter values are shown in Table 1.

**Table 1:** The model parameter values.

Parameter	Value	References
*a*	25 ± 1.25	Cunha et al., 2022, 2023
*b*	30 ± 1.5	Cunha et al., 2022, 2023
*c*_(*j*)_	$\frac{5.10^{-2}}{d.|i-j|}$	Cunha et al., 2022, 2023; Anastassiou et al., 2011; Logothetis et al., 2007
*d*	50μ*m*	Cunha et al., 2022, 2023; Anastassiou et al., 2011
τ	6 ms	Roth and van Rossum, 2009; Gerstner et al., 2014

### 2.3 Network topology

Synaptic connections between neurons are organized according to a small-world topology following the Watts-Strogatz model (Watts and Strogatz, 1998). The network consists of 80% excitatory neurons and 20% inhibitory. Excitatory and inhibitory neurons were randomly assigned at the beginning of each simulation and remained fixed throughout the simulation. Excitatory synapses were assigned positive coupling weights, whereas inhibitory synapses received negative coupling weights. This topology combines efficient information transfer with local specialization (Watts and Strogatz, 1998; Bassett and Gazzaniga, 2011).

In this work, we adopt the same topological parameters employed in recent studies investigating changes in network complexity associated with communication equilibrium rearrangements (Moreno Cunha et al., 2024). The initial synaptic network was generated as a regular one-dimensional ring lattice in which each neuron was connected to its nearest neighbors. The parameter *nb* denotes the total number of synaptic neighbors connected to each neuron (corresponding to *nb*/2 neighbors on each side of the ring), and simulations were performed for *nb*∈4, 8, 16. The rewiring probability was varied over a broad range, from 0% to 100% (*rp*∈[0, 100]%), allowing the network to interpolate between regular, small-world, and random configurations. Each edge of the initial ring lattice was independently rewired with probability *rp*, following the original Watts-Strogatz algorithm. During the rewiring procedure, self-connections and duplicated edges were not allowed. After rewiring, the adjacency matrix was symmetrized, resulting in an undirected network. The inhibitory connections were assigned randomly by multiplying the corresponding synaptic weights by −1.

While synaptic interactions follow a small-world structure, electric interactions are modeled as an all-to-all weighted network. In this case, the coupling strength depends on the effective distance between neurons, consistent with the reduced electrical coupling formulation adopted in the QIF-E model. As a result, the network incorporates two distinct communication mechanisms (structured synaptic connectivity and global electrical coupling), whose interplay influences the dynamical equilibrium of the system (Moreno Cunha et al., 2024).

To evaluate the statistical variability associated with the network topology, each simulation was performed using an independently generated Watts-Strogatz network. The only source of variability between simulations is the random realization of the rewiring process, while all neuronal, synaptic, and electrophysiological parameters remain unchanged. Consequently, the ensemble of simulations represents statistical fluctuations around the same prescribed network topology rather than different neuronal models or parameter sets.

The output signal obtained from the simulations is the Local Average Potential (LAP), defined as the average membrane potential across all neurons in the network. The LAP is computed as


$$LAP\left(t\right)=\frac{1}{N}\overset{N}{\underset{i=1}{\sum }}V_{m}^{\left(i\right)}\left(t\right),$$

where $V_{m}^{\left(i\right)}\left(t\right)$ denotes the membrane potential of the neuron *i*. The membrane potentials are obtained via Euler integration of the coupled Equation 1. In the present study, the LAP is used as a macroscopic observable to assess the impact of connectivity changes on the collective dynamics of QIF-E networks.

The network size was fixed at *N* = 100 neurons throughout the study in order to isolate the effects of electrical coupling while maintaining the same computational framework previously validated in Moreno Cunha et al. (2024). The variability reported in this work corresponds to independent network realizations rather than finite-size fluctuations within individual networks. The electrophysiological parameters used in Equation 1 are summarized in Table 1. Variations in network connectivity were introduced through changes in synaptic and electrical coupling parameters, including synaptic strength ω^(*k*)^, rewiring probability *rp*, number of neighbors *nb*, and electrical coupling weight *c*_(*j*)_.

Synaptic strength was set to represent weak (ω^(*k*)^ = 5) and strong (ω^(*k*)^ = 30) coupling regimes (Nobukawa et al., 2019; Moreno Cunha et al., 2024). The rewiring probability ranged from *rp* = 10% to *rp* = 90% (Watts and Strogatz, 1998; Moreno Cunha et al., 2024). Electric interactions were considered either inactive (ephaptic OFF, *c*_(*j*)_ = 0) or active (ephaptic ON), with coupling strengths within the interval reported in Table 1. Numerical integration was carried out using MATLAB^®^, employing the Euler method with a time step of 0.001, corresponding to 1ms.

The parameter values were adopted from Moreno Cunha et al. (2024), a exploratory study which provide the parameter analysis to QIF-E model. Therefore, the present study use the knowledge provided by previous one, to limit the parameters space and perform a empirical-model match.

The complete QIF-E simulation code used in this study is available in Supplementary material, allowing the computational experiments presented in this manuscript to be reproduced.

### 2.4 Multiscale entropy

Entropy-based metrics are widely used to quantify irregularity and complexity in dynamical systems (Shannon, 1948; Zhang, 1991; Costa et al., 2002; Richman and Moorman, 2000; Humeau-Heurtier, 2015). Sample entropy (*S*_*E*_) is a robust and widely applied metric, particularly suitable for finite time series (*x*_*i*_).^1^ It is designed to be relatively insensitive to the length of the series and has been extensively used in physiological data analysis (Richman and Moorman, 2000; Costa et al., 2002, 2008; Humeau-Heurtier, 2015). Sample entropy is associated with time-series regularity and is defined in Equation 2 as (Richman and Moorman, 2000; Chenxi et al., 2016; Costa et al., 2002, 2008; Humeau-Heurtier, 2015):


$$\begin{array}{l} S_{E}\left(m,r,M;\tau \right)=-\ln\left(\frac{\sum_{i=1}^{M-m}n_{i}^{m+1}}{\sum_{i=1}^{M-m}n_{i}^{m}}\right), \end{array}$$ (2)

where $n_{i}^{m}$ denotes the number of template vectors of length *m* that match within a tolerance *r* in the time series *x*_*i*_ (Costa et al., 2002, 2008; Richman and Moorman, 2000; Humeau-Heurtier, 2015). In this study, the embedding dimension was fixed at *m* = 2, and the tolerance was set to *r* = 0.2σ, where σ denotes the standard deviation of the analyzed time series, following standard practice in physiological signal analysis (Costa et al., 2002, 2008; Humeau-Heurtier, 2015).

A known limitation of single-scale entropy measures is their sensitivity to stochastic fluctuations and observational noise, which may lead to high entropy values even for signals with poor informational structure (Costa et al., 2002, 2008; Humeau-Heurtier, 2015). To address this limitation, entropy is evaluated across multiple temporal scales, allowing the assessment of how information content is preserved or reorganized along the time series. This approach is known as Multiscale Entropy (MSE) (Costa et al., 2002, 2008; Humeau-Heurtier, 2015).

For MSE computation, a coarse-graining procedure is applied, in which the original time series is transformed into a set of surrogate time series by averaging non-overlapping segments of length τ (Costa et al., 2002, 2008; Humeau-Heurtier, 2015). Specifically, the coarse-grained series is defined as


$$y_{j}^{\left(\tau \right)}=\frac{1}{\tau }\overset{j\tau }{\underset{i=\left(j-1\right)\tau +1}{\sum }}x_{i},$$

where τ is the scale factor. Sample entropy is then computed for each coarse-grained time series, yielding an entropy profile across multiple temporal scales.

#### 2.4.1 Multiscale entropy for experimental EEG data

The MSE was computed for the EEG time series recorded at each electrode for each participant. The analysis was performed using segments of 1,280 points extracted from each preprocessed signal, and the resulting MSE values were averaged across segments. Sample entropy was estimated for scale factors ranging from τ = 1 to τ = 6. The upper scale limit was chosen based on the sampling frequency of the EEG signals (256 Hz) and corresponds to the range in which MSE exhibits reliable performance for physiological data (Costa et al., 2002, 2005, 2008). Considering the EEG sampling frequency (256 Hz), the scale factors τ = 1–6 correspond to progressively larger temporal windows obtained through coarse-graining. Therefore, the MSE profile reflects changes in temporal organization across increasing observation scales rather than across distinct frequency bands. This procedure enabled the characterization of EEG signal complexity across multiple temporal scales.

For statistical comparison, the per-electrode MSE values were subsequently grouped into five cortical regions (frontal, central-parietal, occipital, left temporal, and right temporal). Regional aggregation was adopted because the limited spatial resolution of scalp EEG, together with volume conduction, renders neighboring electrodes strongly correlated; grouping electrodes into regions therefore produces entropy estimates that are less susceptible to artifacts, reduces the number of statistical comparisons, and matches the spatially coarse-grained level of description of the model observable (LAP).

#### 2.4.2 Multiscale entropy for simulated neural networks

The MSE was also computed for the temporal series obtained from the network simulations. In this study, 30 simulations were performed for each group (ON and OFF) in order to statistically approximate the experiment. For each simulation, a time series of 71,000 points was generated, and the first 1,000 points were discarded to eliminate transient dynamics. The remaining signal was divided into segments of 2,000 points, over which sample entropy was calculated and subsequently averaged. As in the experimental analysis, scale factors from τ = 1 to τ = 6 were considered, ensuring methodological consistency. As in the experimental analysis, scale factors from τ = 1 to τ = 6 were adopted in order to apply the same MSE methodology to both datasets. However, because the simulated signals were generated with a numerical integration time step of 1 ms, whereas the EEG recordings were sampled at 256 Hz, identical values of τ correspond to different physical time windows in the two datasets. Consequently, the comparison performed in this work is phenomenological rather than a point-by-point temporal correspondence. The objective is to compare how MSE changes as a function of coarse-graining within each system, rather than to establish equivalence between identical physical time scales.

#### 2.4.3 Log-Variance Ratio (LVR)

A stable and scale-invariant metric of inter-individual variability across conditions, after compute the standard deviations (see Table 2), is the Log-Variance Ratio (LVR). Considering the standard deviation, σ, of the MSE values within each group, the LVR is defined in Equation 3, as follows:


$$\begin{array}{l} \text{LVR}=2\log\left(\frac{\sigma_{\text{Experiment}}}{\sigma_{\text{Control}}}\right), \end{array}$$ (3)

where σ_Experiment_ and σ_Control_ denote the square of the group standard deviation of the Experimental and Control groups. The LVR provides a symmetric measure centered at zero, where negative values indicate reduced variability in the Experimental group relative to controls, and positive values indicate increased variability (Senior et al., 2020).

**Table 2:** Standard Deviation Ratio to MSE.

τ	Group	Brain region					Group	Model (*nb* = 8)	
		CP	O	RT	LT	F		ω^(*k*)^ = 5	ω^(*k*)^ = 5
								*rp* = 10*%*	*rp* = 90*%*
1	σ_*CG*_	0.148	0.108	0.167	0.133	0.157	σ_*ON*_	0.023	0.006
6		0.265	0.115	0.136	0.148	0.196		0.072	0.022
1	σ_*MG*_	0.052	0.046	0.044	0.049	0.052	σ_*OFF*_	0.007	0.006
6		0.106	0.122	0.079	0.106	0.063		0.021	0.015
1	$\frac{\sigma_{MG}}{\sigma_{CG}}$	0.350	0.428	0.265	0.367	0.329	$\frac{\sigma_{OFF}}{\sigma_{ON}}$	0.304	0.988
6		0.401	1.061	0.584	0.718	0.321		0.292	0.702

MSE Standard Deviation to scales (τ) equals 1 and 6 for CG and MG, beyond the proportion (ratio) between both groups. In addition, the same information to QIF-E model.

The use of the logarithmic transformation improves interpretability and comparability across scales, particularly in regimes where the standard deviation differences are multiplicative rather than additive. In contrast to Variance Ratios, the LVR is less sensitive to scale-dependent distortions and provides a more linear representation of relative dispersion changes across MSE scales, brain regions and model conditions (Senior et al., 2020).

## 3 Results

**Figure 1:**
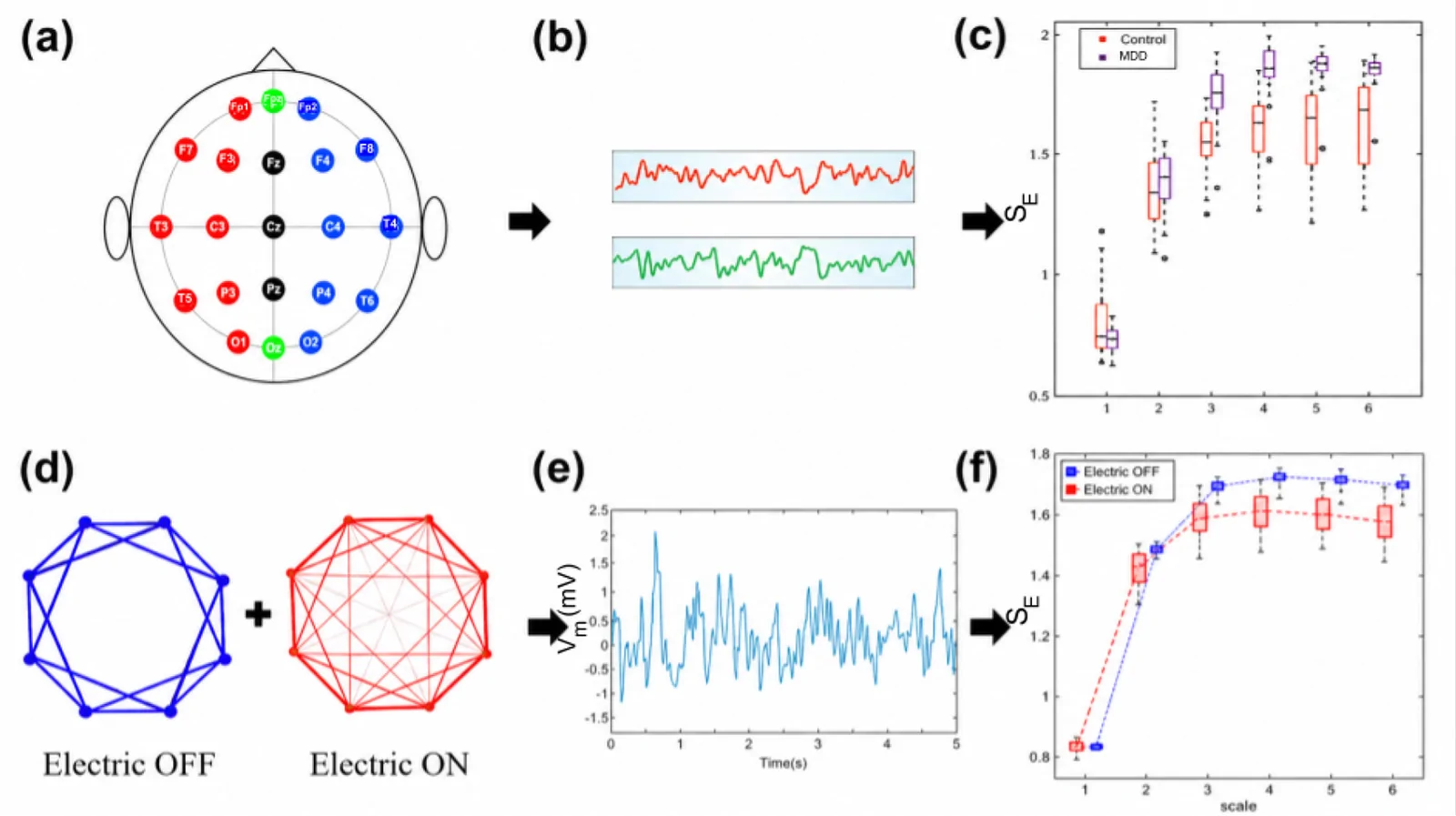
Comparison of multiscale entropy in real and simulated neural networks with ephaptic coupling dynamics. **(a)** The 10–20 electrode placement model used for EEG signal acquisition. **(b)** Representative EEG temporal signals recorded from selected brain regions depicted in **(a)**. **(c)** MSE analysis of the EEG temporal signals, comparing the CG and the MG. **(d)** Schematic illustration of the neuronal network model based on a small-world synaptic topology combined with a distance-weighted ephaptic coupling layer, allowing independent control of synaptic and ephaptic interactions. **(e)** LAP obtained from the simulated network, defined as the spatial average of the membrane potentials across all neurons. **(f)** MSE profiles obtained from the simulated networks under ephaptic ON and ephaptic OFF conditions, shown alongside experimental EEG results to enable a qualitative comparison of complexity patterns across scales.

Figure 1 presents a qualitative comparison between the MSE profiles obtained from empirical EEG signals and simulated neuronal network dynamics. Panel (a) illustrates the electrode positioning according to the international 10–20 system used for EEG acquisition. Representative EEG signals recorded from selected brain regions are shown in panel (b), illustrating the temporal variability of the neural activity across channels.

In panel (c), the MSE of EEG signals is shown for the control group (CG) and the major depressive disorder group (MDD). The MSE profiles indicate that, although both groups exhibit scale-dependent changes in entropy, the MDD group shows a systematically reduced dispersion of entropy values across scales when compared to the CG. This may be consistent with a more homogeneous MSE profiles in the MG.

To investigate whether simplified neuronal network models can phenomenologically reproduce qualitative patterns of complexity similar to those observed experimentally, we analyzed a computational neural network based on a small-world synaptic topology combined with an additional local electrical coupling layer, as schematically illustrated in panel (d).

In this model, we simulated two distinct scenarios: a network with local electrical coupling enabled (denoted as 'Electric ON') and a network in which the coupling term was suppressed ('Electric OFF'). The resulting network activity was summarized using the Local Average Potential (LAP), shown in panel (e), which provides a coarse-grained representation of the collective network dynamics.

Panel (f) displays the MSE profiles obtained from the simulated networks. A qualitative comparison with the experimental EEG results reveals that networks lacking local electrical coupling exhibit entropy profiles with reduced dispersion across scales, qualitatively resembling the patterns observed in the MDD group. These findings do not establish a causal link between electrical field effects and depression; instead, they indicate that alterations in generalized coupling mechanisms can lead to network dynamics with constrained complexity, consistent with experimental observations.

### 3.1 Empirical EEG analysis

**Figure 2:**
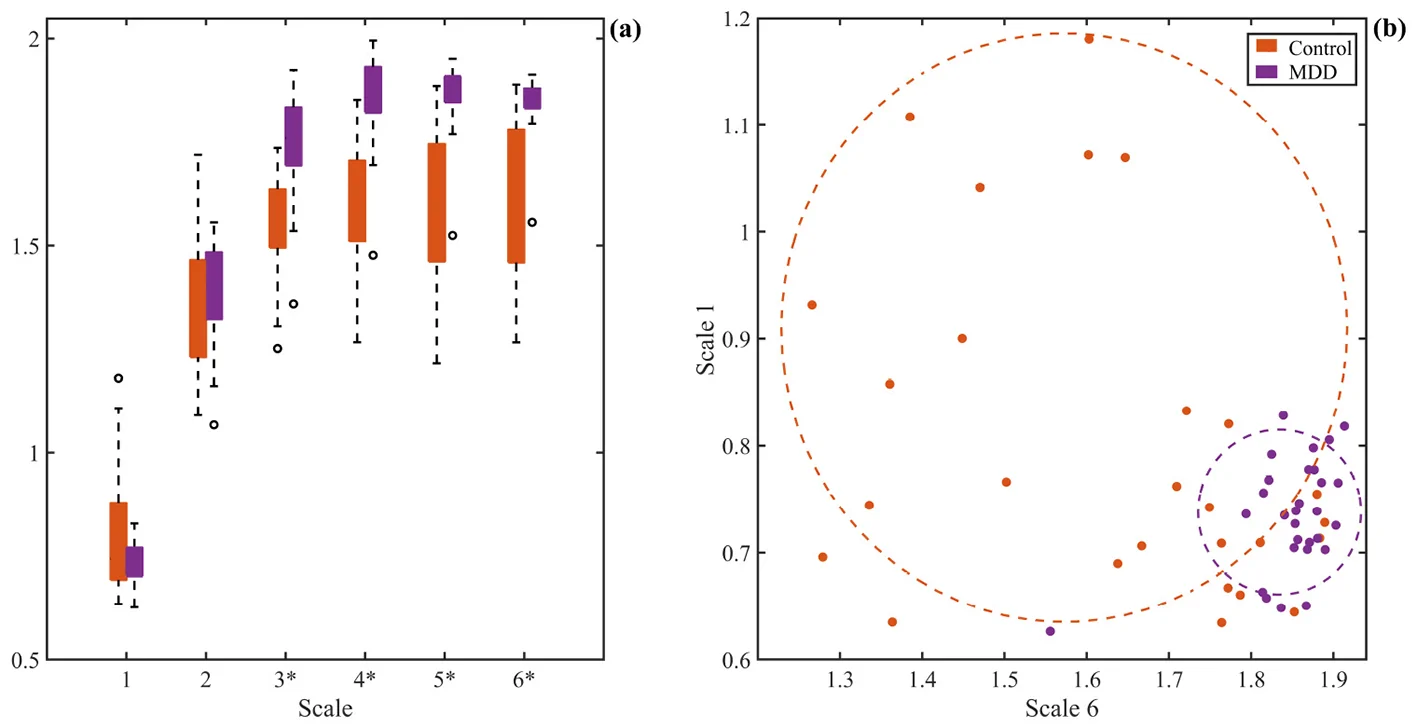
Multiscale entropy analysis in MG and CG. **(a)** Shows the MSE of the EEG signals of the group of patients with depression (purple) and the CG (orange) on scales 1–6. A monotonous growth of the mean entropy from scale 1 to scale 6 is noted in both groups, with the mean entropy of the MG being higher, for scales above 3, compared to the CG. **(b)** Illustrates the dispersion of the correlation between the entropy on scales 1 and 6 for the two groups. The CG presents greater dispersion of entropies, without a clear correlation between the scales, while the MG group showed greater concentration of values, indicating less dispersion and absence of significant correlation.

MSE analysis was conducted to compare the control group (CG) and the MDD group (MG) on scales 1–6 (Figure 2). In Figure 2a, a monotonic increase in mean entropy is observed from scale 1 to scale 6 in both groups. However, from scale 3 onward, the mean entropy values of the MG are significantly higher than those of the CG (*p* = 0.008 obtained by Bonferroni multiple comparison correction across scales; see Table 3). This indicates higher signal irregularity for patients with depression at wider time scales. Conversely, at scale 1, no statistically significant difference was found, indicating that signal complexity is comparable between groups at shorter time scales.

**Table 3:** Significant differences between MG and CG of MSE.

	Brain region				
Scale	LF	CP	O	LT	RT
1	0.038	0.052	0.045	0.021	0.037
2	0.46	0.377	0.792	0.949	0.823
3	0.0	0.0	0.047	0.0	0.02
4	0.0	0.0	0.0	0.0	0.0
5	0.0	0.0	0.0	0.0	0.0
6	0.0	0.0	0.017	0.0	0.0

The table demonstrates significant differences across all analyzed brain regions, as determined by Analysis of Variance (ANOVA), with a significance level of 0.008. Specifically, the depression group exhibits higher entropy values in all regions at scales 4 and 5. At scale 6, however, the depression group does not show a significant increase in entropy in the occipital region.

In addition to the differences in mean values, the groups presented distinct patterns of data dispersion. The CG exhibits greater inter-individual variability in entropy across all scales, as indicated by the larger interquartile range in Figure 2a. This greater dispersion suggests a higher degree of dynamical heterogeneity among healthy individuals. In contrast, the MG presents narrower dispersion, indicating greater homogeneity in the patterns of neuronal variability among the participants, despite displaying higher overall complexity.

**Figure 3:**
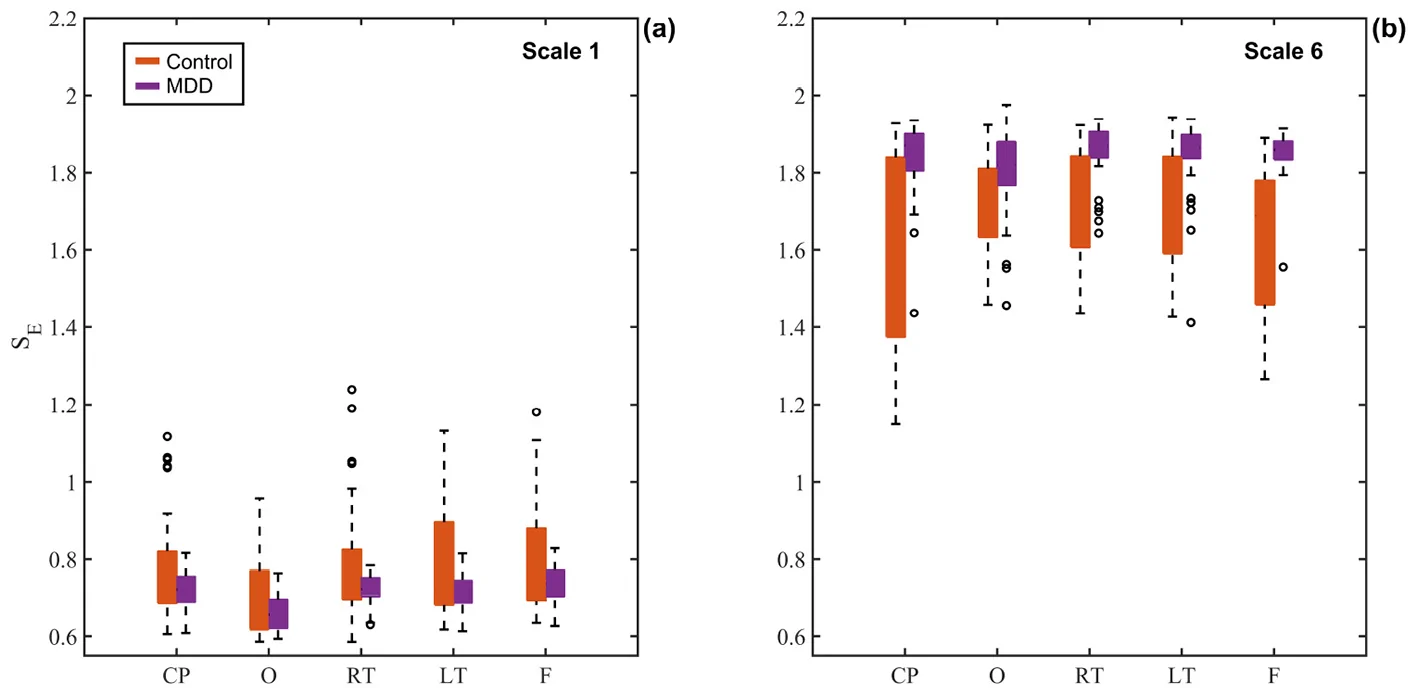
Analysis of the entropy of EEG signals in different brain regions for the CG and MG. **(a)** Presents the MSE on scale 1 (S1) for all cortical brain regions analyzed: frontal (F), left temporal (LT), right temporal (RT), occipital (O) and central parietal (CP). It can be observed that the mean entropy between the MG and the CG didn't present a significant difference for all brain regions. However, the CG exhibits greater entropy dispersion between individuals, suggesting greater variability in neuronal complexity patterns. **(b)** Shows the mean entropy values on scale 6 (S6) for both groups, evidencing an increase in entropy in relation to scale 1, indicating greater complexity of neural signals on broader temporal scales. The comparison between the groups revealed that the MG presents a higher mean entropy than the CG in all regions analyzed. However, the CG maintains a greater variability of entropy between individuals, reflecting a greater dynamic diversity in the EEG within this group. This difference between the groups is statistically significant for all regions, except for the occipital region, where no significant difference is observed in the S6 scale.

Figure 3 presents the entropy analysis on scales 1 and 6 of the EEG signals for the CG and MG in different cortical regions. In panel (a), we observed the distribution of entropy values on scale 1 (S1) for the frontal (F), left temporal (LT), right temporal (RT), occipital (O) and central parietal (CP) cortical regions. There is no statistically significant difference between the mean entropy values between the two groups on this scale 1. However, the CG presents a greater dispersion of entropy values, indicating greater variability in the complexity of the neural signals of the individuals in this group.

In panel (b), we analyze the mean values and dispersion of entropy on scale 6 (S6) for all brain regions analyzed, evidencing a generalized increase in entropy compared to scale 1. This increase suggests greater dynamic complexity of neural signals on broader temporal scales. Furthermore, the MG presented higher mean entropy values than the CG in all regions analyzed. Despite this, the CG continues to exhibit greater inter-individual variability, reflecting a broader dynamic diversity in brain activity patterns.

### 3.2 Computational model results

**Figure 4:**
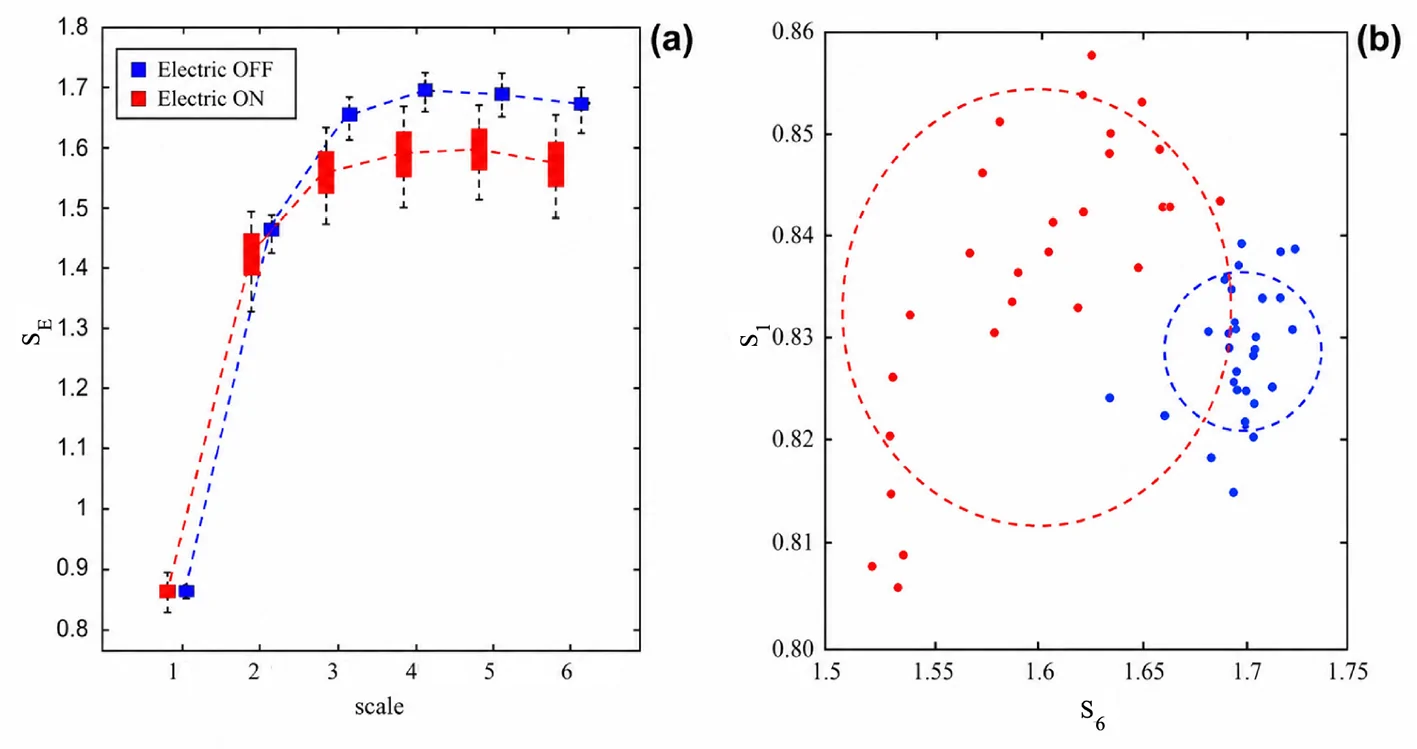
Multiscale entropy analysis in neural networks simulated with and without ephaptic coupling. **(a)** Average MSE results (scales 1–6) for 30 simulations of a neural network with 100 neurons, 8 neighbor and 10% of re-connection: electric coupling off (blue) and electric coupling on (red). Both groups showed a monotonous growth of entropy across scales, as also observed in the experimental EEG data (CG and MG). With electric coupling on, a reduced average entropy was observed, but with greater dispersion between simulations, indicating greater individual variability. When electric coupling is off, the average entropy was higher at scales above 3, suggesting a more erratic and less controlled dynamics, but more homogeneous (less dispersion) among simulations of the same group. **(b)** Correlation and dispersion of data for each simulation in both groups: electric coupling on and electric coupling off. The results indicate that electric coupling contributes to a richer and more varied dynamics in the system, supporting a broader and more stable inter-simulation variability, despite a lower average complexity.

Figure 4 presents the MSE analysis of the Local Average Potential (LAP) from simulations of a neural network (*N* = 100), comparing scenarios with active and inactive local electrical coupling. Panel (a) shows that both configurations demonstrate monotonic entropy growth across scales. However, in simulations with active coupling (red line), the average entropy is lower, while the dispersion between simulations is greater. This behavior phenomenologically aligns with the CG EEG data. On the other hand, when electrical coupling is inactive (blue line), the average entropy increases on scales above 3, and a smaller dispersion is observed between realizations. This pattern parallels the results observed in the MDD group (MG). Panel (b) illustrates the correlation and dispersion for each simulation. The results indicate that local electrical interaction enriches the system's dynamics, promoting greater variability and balance (greater dispersion and lower average entropy), suggesting that such mechanisms may be essential for maintaining a diverse range of dynamical states.

### 3.3 Topological and parametric influence

**Figure 5:**
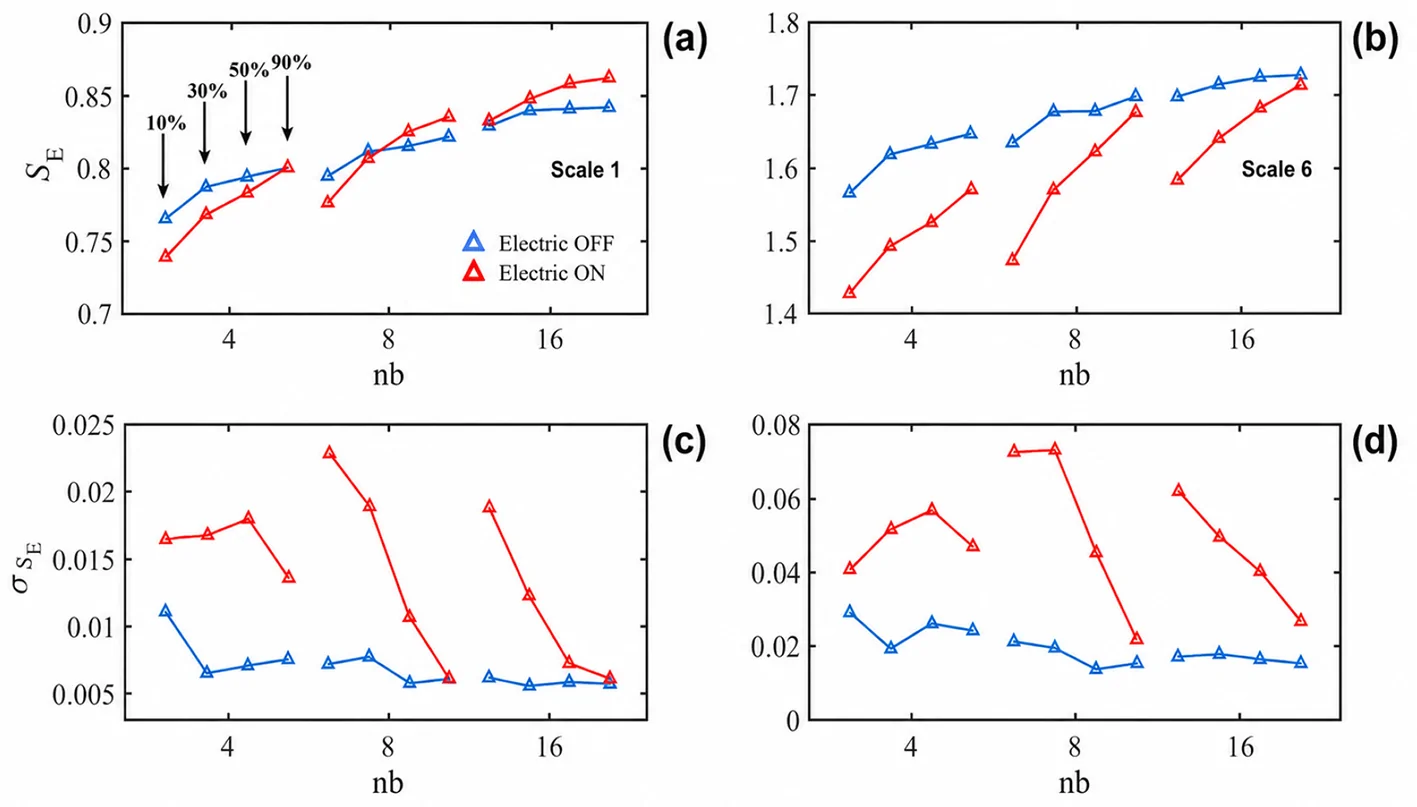
Effect of electric coupling and synaptic strength on the mean entropy and variability of a neural network. **(a)** Mean entropy at scale S1 for the two simulated conditions: electric coupling ON (red) and electric coupling OFF (blue, synaptic coupling only). The x-axis represents topological changes in the network, combining variations in rewiring probability (10%, 30%, 50%, and 90%, indicated by arrows) with different numbers of first neighbors (*nb* = 2, 4, 8). An overall increase in mean entropy is associated with higher rewiring probabilities and a larger number of first neighbors, reflecting the influence of network topology and synaptic structure on short-scale dynamics, while ephaptic coupling does not induce a systematic separation in mean entropy at this scale. **(b)** Mean entropy at scale S6. A separation between the ephaptic ON and OFF conditions is observed mainly for low rewiring probabilities, indicating that ephaptic coupling modulates the long-scale temporal structure of the network activity. **(c)** Dispersion (standard deviation) of the mean entropy at scale S1. The largest variability occurs for networks with a reduced number of first neighbors and low rewiring probability, characteristic of a small-world regime. Notably, the activation of ephaptic coupling consistently amplifies the dispersion of entropy values, indicating enhanced inter-realization variability in the network dynamics. **(d)** Dispersion of the mean entropy at scale S6. The highest variability is observed for weak synaptic coupling, low rewiring probability, and activated ephaptic coupling, highlighting the role of effective field-mediated interactions in sustaining a broader range of dynamical states at longer temporal scales.

Figure 5 explores how variations in network topology, synaptic strength, and electrical coupling affect entropy. At scale S1 (Panel a), increased randomization (rewiring probability) and higher connectivity (number of neighbors) promote higher signal irregularity. However, the presence of electrical coupling does not produce a systematic separation at this short scale.

Differences between ON and OFF conditions emerge primarily at larger temporal scales (Scale S6, Panel b). The activation of electrical coupling consistently amplifies the dispersion of entropy values (Panel c and d), particularly in the small-world regime with weak synaptic coupling. These results highlight that field-mediated interactions play a central role in sustaining a broader range of dynamical states, qualitatively resembling the higher inter-individual variability observed in the control EEG group.

The Table 2 shows the standard deviation (STD, σ) for τ equals 1 and 6, for CG and MG in experimental measures, besides the σ for ephaptic ON and OFF in the model simulations. In the experimental set are shown the MSE STD to each region in the EEG data, to both scales present in the Figure 2b. Note, in Table 2 the MSE STD is lower for the MG if compared with the CG in all EEG regions. Further, the MSE STD are shown for model results. In Table 2, the two columns on the right side exhibit the STD values for ω^(*k*)^ = 5 with rewiring probability (*rp*) equal 10%, and show STD values for *rp* = 10% and 90% to ω^(*k*)^ = 5. Since it is not possible to compare the experimental with model absolute values, the Variance Ratio was estimated. Therefore, in the last row, Table 2 presents the Variance Ratio(VR-proportion between the MDD STD and the Control STD-experimental; or Ephaptic OFF STD and Ephaptic ON STD-model). For experimental data, the VR in scale 1 lies between 0.265 and 0.428, i.e., the MSE dispersion presents an inter-individual reduction in both scales. However, to scale 6 the results change in the occipital region, which shows the VR greater than 1. The greater values in VR for scale 6 are compatible with the model VR values which are presented in the last right columns.

In Table 4, the LVR values are shown to analyse which model conditions exhibits the best match description when compared to the empirical data. Interestingly, in the empirical results, the brain regions with the consistent highest absolute values of LVR are presented in CP and F regions. On other hand, the condition changes on the model which shows similar LVR absolute values are the electric changes (OFF vs ON) with *rp* = 10%, but not when *rp* = 90%. However, changes in the network topology does not exhibits same LVR under Electric OFF condition. Otherwise, the Electric ON condition shows good match with the empirical data. When we compare the OFF vs ON and the ON vs ON with *p* changes, we see that the OFF vs ON can describe the variance change better than ON vs ON.

Table 4Log-Variance Ratio (LVR).

**Table:** 

Scale (τ)	Brain regions				
	CP	O	RT	LT	F
1	–2.101	–1.699	–2.657	–2.006	–2.225
6	–1.829	0.117	–1.077	–0.664	–2.274

**Table:** 

Model comparison	LVR	
	Model condition	LVR
OFF vs. ON	(τ = 1, *rp* = 10%)	−2.379
OFF vs. ON	(τ = 1, *rp* = 90%)	0.000
OFF vs. ON	(τ = 6, *rp* = 10%)	−2.464
OFF vs. ON	(τ = 6, *rp* = 90%)	−0.766
OFF (varying *rp*)	τ = 1	−0.308
OFF (varying *rp*)	τ = 6	−0.673
ON (varying *rp*)	τ = 1	−2.688
ON (varying *rp*)	τ = 6	−2.371

LVR between MDD and Control groups (EEG data), and between model conditions (OFF/ON and topology variations). Negative values indicate reduced variability in MDD or OFF conditions relative to their respective references.

## 4 Discussion

The present work highlights qualitative similarities between the MSE patterns observed in EEG recordings from individuals with MDD and those obtained from simulations using the QIF-E neuronal network model. This study evaluates an *in vivo* experimental EEG dataset against an *in silico* framework that incorporates local electrical coupling, often termed ephaptic interaction. MSE is a well-established method for probing the temporal structure, predictability, and self-similarity of neural signals across scales (Costa et al., 2002, 2005). Rather than implying biological identity between experimental and simulated systems, this parallel analysis provides a qualitative phenomenological framework to interpret how alterations in network interactions may reproduce population-level entropy features observed in MDD.

Empirical results show that mean entropy values differ between the control group (CG) and the MDD group (MG), with this distinction being more pronounced at higher scales, particularly at scale S6 (Heisz and McIntosh, 2013). The increase in entropy at larger scales in the MG indicates that on average, the EEG signals exhibit enhanced long-range temporal irregularity compared to the CG (Liu et al., 2024). Importantly, an increase in entropy at specific scales should not be interpreted as a simple marker of pathology. Instead, it may reflect a distinct form of neural organization, where predictability is reduced while structured temporal dependencies are preserved across longer scales.

Neuroimaging studies provide a plausible neurobiological substrate for these observations. Reported volumetric reductions in frontal regions in individuals with MDD (Frodl et al., 2008) could potentially influence large-scale neural coordination, thereby affecting the temporal organization of EEG signals. From a neurochemical perspective, alterations in glutamatergic and GABAergic balance are well-documented in depression (Duman et al., 2019). Such dysregulation is consistent with the observed increase in entropy at higher scales, supporting the idea that altered local regulation can propagate to macroscopic EEG dynamics (Breakspear, 2017).

A key result of this study concerns the inter-individual dispersion of entropy values. As shown in Figure 2, the CG exhibits a larger dispersion of mean entropy, whereas the MG displays reduced dispersion. This suggests that, although the MG signals are more “complex” (higher entropy), the patients themselves are more “similar” to each other in their dynamical profiles. This reduced dispersion indicates that individuals with MDD converge toward a more homogenous dynamical regime, leading to a loss of inter-individual heterogeneity across the population when compared to healthy controls.

Simulation results provide further insight from a computational perspective. Networks lacking local electrical coupling exhibited higher mean entropy but reduced dispersion across realizations, whereas networks with active electrical coupling showed increased dispersion despite slightly lower mean entropy (Figure 4). These findings indicate that generalized local field interactions modulate the variability of MSE across network realizations. Moreover, our results suggest that the QIF-E framework, despite its structural limitations, is capable of reproducing qualitative features observed in neural dynamics.

Comparing simulated and experimental results reveals a notable qualitative correspondence. The reduced inter-realization dispersion observed in coupling-OFF simulations parallels the entropy patterns found in the MG, whereas the increased dispersion induced by electrical coupling mirrors the broader variability observed in the CG. This suggests that the model expand the accessible inter-simulation variability of the network by the Local Electrical Coupling, increasing inter-realization entropy variability without necessarily maximizing mean entropy.

Rather than implying biological identity between experimental and simulated systems, this parallel analysis provides a qualitative phenomenological framework to interpret how alterations in network interactions may reproduce population-level entropy features observed in MDD.

### 4.1 Limitations and perspectives

One limitation of the present study is that the publicly available EEG dataset provides only limited clinical characterization of the participants. Consequently, important variables such as disease duration, recurrence status, comorbid psychiatric conditions, sleep quality, educational level and medication history were not available for analysis. Because these clinical factors may influence neural dynamics, they could also contributes to the inter-individual variability observed in multiscale entropy measurements. Therefore, future studies using clinically better-characterized cohorts will be important to determine the extent to which these factors contributes to the entropy patterns reported in the present study.

The QIF-E model is intentionally simplified and does not account for morphological details (Shneider and Pekker, 2015). Critically, the present study does not establish ephaptic dysfunction as a causal mechanism in MDD. Instead, it demonstrates that incorporating simplified ephaptic interactions enables a closer qualitative match between simulated and experimental population-level entropy patterns. Additionally, although the QIF-E model is not a macroscopic framework, the observed entropy correspondence suggests that the model captures relevant multiscale dynamical properties of neural systems. Future studies should investigate whether these model-based observations translate to specific physiological disruptions in non-synaptic communication. Moreover, future work should explore changes to other model parameters to determine whether the electric field is the unique mechanism capable of qualitatively reproducing the experimental features in the MSE.

### 4.2 Conclusion

This study investigated brain signal complexity in Major Depressive Disorder through a phenomenological population-level analysis of MSE. The experimental results revealed that, although individuals with MDD exhibit higher entropy at larger temporal scales, they also show a marked reduction in inter-individual dispersion.

Computational simulations provided a theoretical framework to interpret these observations. Networks with suppressed local electrical interactions reproduced entropy patterns qualitatively similar to those observed in the MDD group. In contrast, the inclusion of field-mediated interactions consistently increased dispersion across network realizations, mimicking the broader variability found in healthy controls.

Importantly, this work does not claim a causal link between altered electrical coupling and depression. Instead, it demonstrates that the QIF-E model offers a plausible dynamical account for experimentally observed entropy patterns. Overall, these findings suggest that non-synaptic interactions warrant further exploration as a complementary mechanism influencing large-scale temporal organization in neural systems.

## Data Availability

The original contributions presented in the study are included in the article/Supplementary material, further inquiries can be directed to the corresponding author.

## Footnotes

1 with the time series denoted by *x*_*i*_∈*x*_1_, *x*_2_, …, *x*_*M*_, where *M* is the length of the time series and *i*∈[1, *M*].
