# Supplementary material for: Reduced dynamical variability in depression: evidence from EEG and QIF-E network simulation
Source: Front Comput Neurosci. 2026 Aug 27;20:1899952. doi: 10.3389/fncom.2026.1899952 (PMC13558199; doi:10.3389/fncom.2026.1899952)
Supplement: Supplementary file 1 [file Data_Sheet_1.pdf]

frontiers

Supplementary Material

EXPERIMENTAL DATASET DEMOGRAPHY

Table S1. Demographic characterization of the MG sample.

**Variable**	**Category**	**n**	**%**
Gender	Feminine	17	51.5
	Masculine	16	48.5
	**Total**	**33**	**100.0**
Age (years)	20–24	8	24.2
	25–29	1	3.0
	30–34	1	3.0
	35–39	4	12.1
	40–44	5	15.2
	45–49	5	15.2
	50–54	3	9.1
	55–59	6	18.2
	**Total**	**33**	**100.0**
Antidepressant classes used	Antipsychotics	2	6.1
	Benzodiazepines	4	12.1
	MT agonists	1	3.0
	SNRI	1	3.0
	SSRI	18	54.5
	Tricyclics	5	15.2
Stop medication		2	6.1

Abbreviations: MT = Melatonin; SNRI = Selective Norepinephrine Reuptake Inhibitors; SSRI = Selective Serotonin Reuptake Inhibitors.

QIF-E MODEL CODE

The QIF-E model code used to the generate all data in the present study is available bellow.

```
% Programme designed to simulate ephatic coupling, using the
% ephatic quadratic integrate-and-fire model.
% Developed on 12/2023 by Gabriel Moreno Cunha.
% Neurodynamic Simulation and Modelling Laboratory-UFRN, Natal-RN-BR
%% ------------
% INPUT PARAMETERS OF THE FUNCTION (IN THE ORDER OF THE INPUT):
```

```
%A. n -> Number of Neurons (Greater than 2)
%B. viz -> Half Number of neighbours
%C. simulate -> Number of simulations for a set of fixed parameters
%(integer value)
%D. t_final -> Total time in seconds
%E. prob -> Probability of reconnection in the synaptic small-world (less than 1)
%F. weight -> synapse intensity
%G. pesoefa -> exponent of ephatic communication (USE 0 IF YOU DON'T WANT TO APPLY)

%% ------------
% OUTPUT PARAMETERS OF THE FUNCTION (IN THE ORDER OF THE OUTPUT WITH DIRECT SAVE):
%A. v -> LFP of the network
% Enjoy of it!
clear all
%Simulation parameters
n = 100; % neuron number
viz=2; % Half Neighbor number
simul=[30]; % Number of simulations
t_final=10; % Each simulation Total Time (s)
prob=.15; % Rewiring probability
peso=5; % Synaptic weight
pesoefa=2; % Ephaptic Exponent (0- ephaptic OFF; 2 - ephaptic ON)


% Numeric Constants
dt = 0.01; % step (dt)
tp = 0:dt:t_final; % time
N_steps = length(tp); % Steps Number


%% Simulation
for zz = simul
    tic
    for pr = 1:length(prob)
        [s,t] = C_sw(n,viz,prob(pr)); % Adjacency Matrix generated by the function (
    Synapses)
        ad1 = zeros(n); % adjacency matrix
        for i = 1:length(s(:,1))
            for o = 1:length(t(1,:))
                if s(i,1)~=t(i,o)
                    ad1(i,t(i,o)) = 1;
                end
            end
        end
```

```
        adj = ad1'+ad1; % Symmetric Matrix
        f=randi([1 n],1,.2*n); % Inhibitory population
        adj(f,:)=-adj(f,:); % Indexing the Inhibitory on the adjacency matrix
        for syn = 1:1:length(peso)
            for y = 1:length(pesoefa)
                v = zeros(1,N_steps); % LFP Vector
                x = zeros(n,N_steps); % Neurons Time series
                %% defining communications: Topology area
                g = zeros(n,N_steps); % Count Synaptic Inputs
                fired = zeros(n,N_steps); % Spikes Graphic
                gamma = peso(syn); % Ephaptic Coupling Intensity.
                fire = zeros(n,N_steps); % Spikes Sum for each neuron

                % Model Constants
                a = linspace(23.75,27.25,n);
                b = linspace(28.5,31.5,n);
                c = C_swe(n); % Adjacency Matrix generated by the function (Ephaptic)
                if pesoefa(y) == 0
                    Amp = 0; % Ephaptic OFF
                else
                    Amp = 5; % Ephaptic ON
                end
                c = c.*Amp*10^(-1*pesoefa(y)); % Ephaptic weight
                I = zeros(n,N_steps); % Ephaptic Sum for each neuron
                % Defining the model and the variables
                f1 = @(tp,g,x,a,b,I) (a*x^2+b*x+9+g-I); % QIF-E equation

                % Initial Conditions
                x0 = zeros(1,n); % Initial Conditions for each neuron
                tic % Simulation Time Count (MATLAB FUNCTION)
                % For each neuron
                for i=1:N_steps-1
                    x(:,1) = x0(:); % Assigning Initial Conditions
                    g = (gamma*(mtimes(adj,fire))); % Synpatic Inputs Sum
                    %% Euler Integration
                    for k = 1:n
                        for h = 1:n
                            if k ~= h
                                I(k,i) = I(k,i)+(c(k,h)*(x(k,i)-x(h,i))); % Ephaptic
    Coupling Sum (Superposition Principle)
                            else
                                I(k,i) = I(k,i); % Ephaptic Coupling Sum
                            end
                        end
```

```
                        x(k,i+1) = x(k,i) + f1(tp(i),g(k,i),x(k,i),a(k),b(k),I(k,i))*dt; % Euler Integration
                        %% Reset Condition and Synaptic simulation
                        if x(k,i+1) >= 90
                            x(k,i) = 90; % Peak
                            x(k,i+1) = -5; % Hyperpolarization
                            for p = i:i+20
                                fire(k,p) = exp(-(p-i)/6); % Neighbor synaptic function
                            end
                            fired(k,i) = k;

                        end
                    end
                end

                %%
                v = mean(x); % LFP
                %% Graphic Section
                figure1 = figure('Color',[1 1 1]);
                plot(tp,v,'-k','LineWidth',2)
                xlabel('Time(s)')
                ylabel('LFP(mV)')
                figure2 = figure('Color',[1 1 1]);
                for q =1:n
                    hold on
                    plot(tp,fired(q,:),'.k','MarkerSize',8)
                end
                xlabel('Time(s)')
                ylabel('# neuron')
                ylim([.2 n+.2])
                xlim([0 t_final])
            end

        end
    end
end

toc


%% Small-World Topology (synaptic communication)
% Copyright 2015 The MathWorks, Inc.

function [s,t] = C_sw(N,K,beta)
```

```
% H = WattsStrogatz(N,K,beta) returns a Watts-Strogatz model graph with N
% nodes, N*K edges, mean node degree 2*K, and rewiring probability beta.
%
% beta = 0 is a ring lattice, and beta = 1 is a random graph.

% Connect each node to its K next and previous neighbors. This constructs
% indices for a ring lattice.
s = repelem((1:N)',1,K);
t = s + repmat(1:K,N,1);
t = mod(t-1,N)+1;

% Rewire the target node of each edge with probability beta
for source=1:N
    switchEdge = rand(K, 1) < beta;

    newTargets = rand(N, 1);
    newTargets(source) = 0;
    newTargets(s(t==source)) = 0;
    newTargets(t(source, ~switchEdge)) = 0;

    [~, ind] = sort(newTargets, 'descend');
    t(source, switchEdge) = ind(1:nnz(switchEdge));
end


end
%% All-to-all weigthed Topology (Ephaptic Coupling)
function [adje] = C_swe(n)

aux = zeros(n);

for j = 1:(n+2)/2
    if 1 ~= j
        aux(1,j)=(1/abs(1-j));
    end
end
for k = n:-1:(n+2)/2
    if 1 ~= k
        aux(1,k) = aux(1,abs(n-k+2));
    end
end


for i = 2:n
```

```
    for l = i:n
        aux(i,l)=aux(i-1,l-1);
    end
end

aux = aux'+aux;
adje=aux;
end
```
